# The Effect of the Timing of Invasive Management on Cardiac Function in Patients with NSTE-ACS, Insights from the OPTIMA-2 Randomized Controlled Trial

**DOI:** 10.3390/jcm10163636

**Published:** 2021-08-17

**Authors:** Nick D. Fagel, Stefan G. J. Leuven, Wouter J. Kikkert, Michelle M. de Leau, Loek van Heerebeek, Robert K. Riezebos

**Affiliations:** Heart Center, Department of Cardiology, OLVG Hospital, 1091 AC Amsterdam, The Netherlands; sgjleuven@gmail.com (S.G.J.L.); w.j.kikkert@olvg.nl (W.J.K.); m.m.deleau@olvg.nl (M.M.d.L.); l.vanheerebeek@olvg.nl (L.v.H.); r.k.riezebos@olvg.nl (R.K.R.)

**Keywords:** acute coronary syndrome, timing, treatment strategy, echocardiography, global longitudinal strain

## Abstract

The timing of coronary angiography in patients with non-ST-elevation acute coronary syndrome (NSTE-ACS) remains a matter of debate. The relationship between the timing of invasive management and left ventricular function (LVF) is largely unknown. The An Immediate or Early Invasive Strategy in Non-ST-Elevation Acute Coronary Syndrome trial (OPTIMA-2) was a randomized controlled prospective open-label multicenter trial that randomized 249 NSTE-ACS patients to either an immediate (<3 h) invasive treatment strategy or an early strategy (12–24 h). Patients were pre-treated with a combination of aspirin, ticagrelor and fondaparinux. The aim of this prespecified sub-analysis was to assess (the recovery of) left ventricular function by analysing echocardiography data obtained <72 h after admission and at 30-day follow-up, for patients with a confirmed diagnosis of acute coronary syndrome. LVF was determined using ejection fraction (EF) and global longitudinal strain (GLS). Inter-observer variability was tested. No difference in the recovery of EF was found between an immediate and early strategy if the follow-up echocardiograms were compared to baseline: 2.5% (standard deviation (SD): 7.9) and 3.3% (SD: 8.5), *p* = 0.51, nor was there any difference in GLS recovery between the study groups: −0.8% (SD: 2.5) vs. −0.7% (SD 2.8) *p* = 0.82. If baseline and follow-up echocardiograms were compared, there was a similar but significant improvement in both EF and GLS in both separate study groups. An immediate invasive strategy in NSTE-ACS patients did not result in an improved left ventricular EF or GLS recovery compared with an early strategy.

## 1. Introduction

For non-ST-segment elevation acute coronary syndromes (NSTE-ACS) a routine invasive strategy is advised according to both the European Society of Cardiology (ESC) and American College of Cardiology (ACC)/American Heart Association (AHA) guidelines [1,2]. In addition, the current ESC guideline recommends early (<24 h) coronary angiography in high-risk acute coronary syndrome (ACS) cases [1]. Several trials have evaluated a variety of different strategies regarding the timing of coronary angiography and the revascularization of clinically stabilized NSTE-ACS patients [3,4,5,6]. The main trials on this subject found no difference in clinical endpoints between patients randomized to early (<14 h) or a delayed invasive strategy (48 to 72 h), except for very high-risk patients.

The “An Immediate or Early Invasive Strategy in Non-ST-Elevation Acute Coronary Syndrome trial” (OPTIMA-2) was designed to study the impact of the timing of an invasive strategy on the total infarct size combining both the effects of spontaneous myocardial infarction and periprocedural-related injury in patients presenting with NSTE-ACS, with the use of modern antiplatelet and anticoagulant therapy. The main results of the OPTIMA-2 have been published previously. In short, no significant difference in infarct size (measured as area under the curve (AUC) of creatine kinase myocardial band (CKMB)) or clinical endpoints (death, non-fatal MI or new revascularization) was found between an immediate (<3 h) and early (12–24 h) invasive strategy [7].

Echocardiography is the most-used imaging modality to assess cardiac function in the acute phase of the NSTE-ACS. In addition, global longitudinal strain (GLS) measurement with speckle tracking echocardiography represents an emerging modality for more accurate assessment of (regional) cardiac function [8,9]. GLS has proven its value as a powerful independent predictor for clinical outcome, including left ventricular (LV) remodeling after coronary angioplasty in patients with recent non-ST elevation myocardial infarction (NSTEMI) [10]. The current study was performed to assess the impact of the timing of the intervention on the recovery of LV function in patients with NSTE-ACS at hospitalization and at follow up.

## 2. Materials and Methods

### 2.1. Study Design

The OPTIMA-2 trial was a prospective, open-label, randomized controlled trial (Netherlands Trial Register identifier: NTR3861) performed by the OLVG hospital in Amsterdam, the Netherlands. The complete study design and the main clinical outcomes have been published previously [7]. In short, patients aged 21 and above with high-risk criteria for NSTE-ACS who had an episode of chest pain within the last 24 h before admission were eligible for inclusion. High-risk criteria were considered: 1 mm of horizontal or downsloping ST depression, dynamic ST- or T- wave changes >1 mm in two contiguous leads, elevated high sensitive-troponin (cardiac Roche high-sensitive TroponinT (hsTroponinT) assay >1× upper limit normal (ULN), defined as >0.014 ug/L), known coronary artery disease, or at least two risk factors: diabetes mellitus, known hypertension, smoking, family history for ischemic heart disease, dyslipidaemia, peripheral artery disease or aged 60 and older. The main exclusion criteria were acute ST-elevation myocardial infarction (STEMI), refractory angina, hemodynamic instability or the use of oral anticoagulants. Eligible patients were randomly assigned to an immediate (<3 h) or early (12–24 h) invasive strategy, defined as the time between admission and angiography. Echocardiographic data were obtained, and the analysis of GLS and ejection fraction (EF) was pre-specified in the study protocol. The study complied with the principles set out in the Declaration of Helsinki. Written informed consent was obtained from each patient. The OPTIMA-2 study was terminated prematurely. Originally it was planned to include 350 patients. However, following an interim analysis after 71% of the anticipated enrolment, the study safety officer concluded that it would be futile to continue enrolment, because of slow patient enrolment in combination with the absence of any signal of improved efficacy of the investigative treatment on the primary endpoint.

### 2.2. Study Procedure

After enrolment, all patients were given ticagrelor and aspirin (loading dose) directly, except those patients already using aspirin and/or a P2Y12 inhibitor at the time of admission. Fondaparinux was administered to all patients without contra-indication.

All patients underwent cardiac ultrasound within 72 h after admission (baseline), and a follow-up echocardiogram was performed after 30-days (follow-up). The method of revascularization after initial angiography (percutaneous coronary intervention (PCI) or coronary artery bypass grafting (CABG)) was at the discretion of the treating physician or the consensus of the hospital’s heart team. The operator was allowed to use iFR, IVUS or OCT if necessary or to decide to perform ad-hoc PCI after index angiography, but this was not mandatory for the study. For this sub-study of the OPTIMA-2 trial, we analysed only the echocardiographic data of patients with a confirmed diagnosis of NSTE-ACS.

### 2.3. Echocardiography

The echocardiographic examinations were performed by experienced sonographers on General Electric (GE) Vivid S6 or Vivid E9 machines (GE Healthcare, Hoevelaken, NL, The Netherlands). The images were analysed offline with General Electric EchoPac (version 112) software by an experienced investigator blinded to clinical baseline data and endpoints. Left ventricular ejection fraction (LVEF) was calculated using Simpson’s biplane method obtained from apical four- and two chamber views.

Speckle tracking 2D analysis was performed in the apical 4-chamber, 2-chamber and the longitudinal long-axis (apical 3-chamber) views. The endocardium of the LV was traced semi automatically and adjusted manually in case the investigator deemed the tracings to be inaccurate. Segmentation was performed according to the AHA 17-segment model. Each apical view covered six segments. The width of the tracings covered the endo-, myo- and epicardium and was adjusted per segment if necessary. GLS was calculated as the mean value of the peak systolic longitudinal strain of each segment at a frame rate of 40–90 frames/second.

### 2.4. Endpoints

There were two primary endpoints: (1) The comparison between an immediate and an early invasive strategy in improvement of LVEF of the 30-day follow-up echocardiogram in comparison to baseline (within 72 h of hospital admission). (2) The comparison between an immediate and an early invasive strategy in the improvement of GLS if the 30-day follow-up echocardiogram was compared to baseline.

Secondary endpoints were differences in LVEF or GLS measurements at baseline and at 30-day follow-up, if both study groups were compared at each specific time point. In addition, for each individual study group separately, the improvement in LVEF or GLS measurement was calculated if the 30-day follow-up was compared to the baseline echocardiogram.

### 2.5. Study Follow-Up

Follow-up consisted of a visit to an outpatient’s clinic and follow-up echocardiography after 30 days of being discharged. If it was not possible to contact a patient, his or her vital status was obtained from the local authorities.

### 2.6. Statistical Analysis

Data were analysed according to the intention-to-treat principle [11]. Statistical analysis was done with SPSS (version 26.0 for Windows, SPSS, Inc., Chicago, IL, USA). Baseline differences were tested with Student’s t test or the Wilcoxon rank-sum test for continuous variables and the chi-square test for categorical variables. The primary outcome was analysed using the Student’s *t*-test, paired *t*-test (in case data was normally distributed) or one-way ANOVA test. In order to assess the correlation between different variables and changes in left ventricular function (LVF), we first analysed the data using univariate linear regression analysis for the following variables: above 60 years of age, diabetes mellitus, hypertension, current smoking, hypercholesterolemia, peripheral arterial disease, family history of ischemic heart disease, several biomarkers (AUC of CKMB, AUC of hsTroponinT, N-terminal-pro hormone BNP (NT-proBNP) and high-sensitive C-reactive protein (hs-CRP), duration of chest pain before admittance, syntax score, number of diseased coronary arteries, treatment strategy (PCI, CABG or conservative), target vessel, total numbers of stents implanted during index procedure and Global Registry of Acute Coronary Events (GRACE)-risk score at index admission. Beta coefficients were calculated with 95% confidence interval (CI) (Supplementary file 1). In case of statistically significant beta coefficients, relevant variables were planned to be included in the multivariate regression model. Tests were 2-tailed, and a value of *p* ≤ 0.05 was considered statistically significant.

Analysis of EF and GLS was done by a single blinded observer. A second observer blindly analysed 20 randomly selected patients and a total of 40 echocardiograms, at the same heartbeat as the first observer. Subsequently, inter-observer reliability was tested by the calculation of the intraclass correlation coefficient (ICC). A two-way mixed model of the absolute agreement type was used. ICC values greater than 0.9 were considered excellent reliability [12].

## 3. Results

### 3.1. Baseline Characteristics

Between March 2013 and November 2018, a total of 249 patients were included in the OPTIMA-2 study. In total, 125 patients were randomized to the immediate invasive group and 124 to the early invasive group. In total, 15 (12%) patients in the immediate group and 12 (10%) patients in the early group ultimately did not have a final diagnosis of NSTE-ACS and were therefore excluded from this analysis (Supplementary file 2). The baseline characteristics of the OPTIMA-2 study have been previously reported and are also summarized in Table 1. The mean age at the time of hospitalization was 65.9 ± 10.5 years in the immediate and 65.8 ± 11.3 years in the early intervention group. No significant differences were found between the two strategy groups in cardiac history and in general cardiovascular risk factors. The average GRACE-risk score was 116.0 ± 27.9 in the immediate and 115.8 ± 28.3 in the early intervention group. Both groups were hospitalized with a median length of 2 days (Interquartile range (IQR) 2.0–4.0).

### 3.2. Echocardiography Data

**Figure 1:**
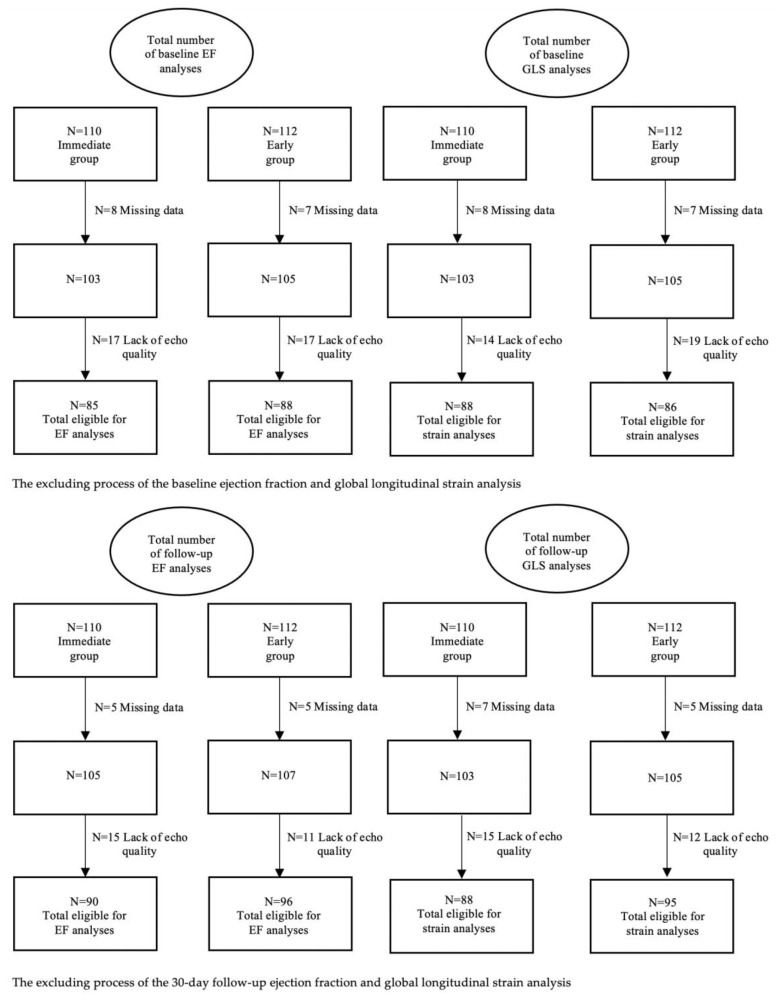
Study flow chart.

In total, 173 (77.8%) baseline echocardiograms were eligible for analysis of EF and 174 (78.4%) GLS. Follow-up echocardiograms were obtained after a median of 31 days (IQR 27–33), after which another 186 (83.8% of total) EF and 183 (82.4%) GLS measurements were included. The patients that were unsuitable for EF calculation due to a lack of echo quality were in most cases also unsuitable for GLS calculation due to the same reason (90% overlap of patients at baseline and 95% at follow-up). The echocardiographic excluding process is shown in Figure 1.

Flow chart of available echocardiography data of participating patients that were randomized to either an immediate invasive strategy or an early invasive strategy.

### 3.3. Endpoints

**Figure 2:**
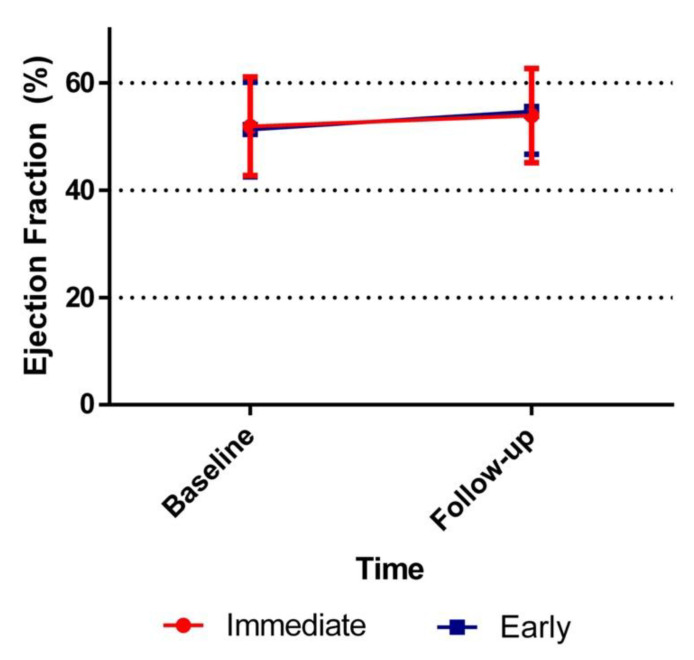
Ejection fraction improvement over time. The ejection fraction of both the immediate invasive strategy and early invasive strategy groups at baseline and at 30-day follow-up.

**Figure 3:**
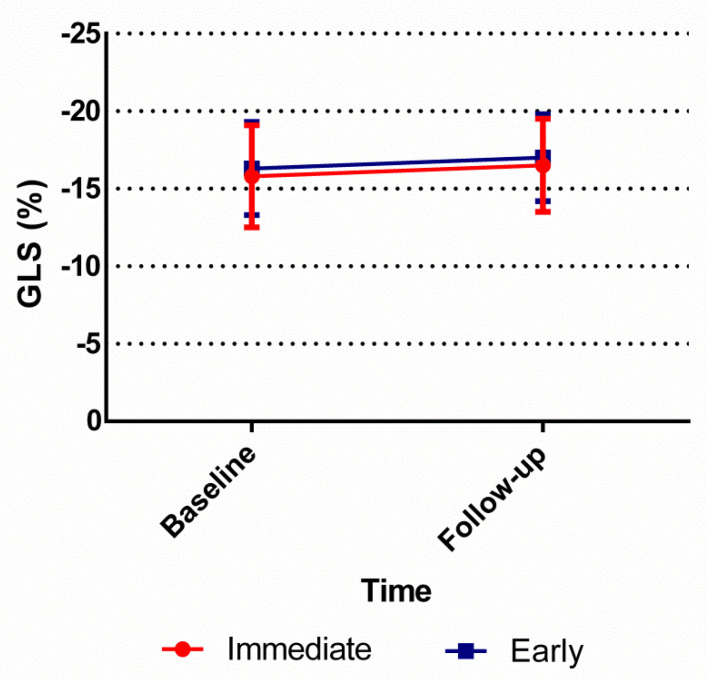
Global longitudinal strain improvement over time. The global longitudinal strain of both the immediate invasive strategy and early invasive strategy groups at baseline and at 30-day follow-up. GLS = global longitudinal strain.

The mean improvement in LVEF after 30 days was similar if an immediate invasive strategy was compared to an early strategy: 2.5%, standard deviation (SD) 7.9 vs. 3.3%, SD 8.5, *p* = 0.51 (Table 2; Figure 2). In addition, no significant difference was found in improvement in GLS if an immediate strategy was compared to an early strategy: −0.8% (SD 2.5) vs. −0.7% (SD 2.8), *p* = 0.82 (Table 3; Figure 3).

If baseline data was compared, we found no difference in LVEF between the study groups, nor did we find any difference between the groups at 30-day follow-up LVEF (Table 2).

When GLS was compared we found similar results: no differences between the study groups at baseline or at 30-day follow-up echocardiogram (Table 3). The LVEF improved significantly if the 30-day follow-up echocardiogram was compared to baseline for both the immediate invasive group (*p* < 0.001) and the early invasive group (*p* < 0.001) separately. In addition, GLS improved significantly if the 30-day follow-up echocardiogram was compared to baseline in the immediate invasive group (*p* < 0.001) and the early invasive group (*p* < 0.001).

By univariate analyses, only NT-proBNP (admission) was statistically significantly associated with change in EF or GLS. The change in EF or GLS for the sub-group of patients treated by PCI were also compared and results are presented in the supplementary files (Supplementary file 3). 

If the different methods of treatment (PCI, CABG or conservative) were compared, we found no significant difference in LVEF improvement at 30-day follow-up (*p* = 0.29), nor in improvement in GLS at 30-day follow-up (*p* = 0.21). For the sub-group of patients treated by PCI, a comparison between the different randomization groups was made regarding improvement in LVEF or GLS. No differences between the immediate (*n* = 43) and early (*n* = 54) invasive groups were observed in LVEF: 2.1% (SD 9.1) vs. 4.6% (SD 8.8), *p* = 0.15) or GLS: −0.8% (SD 2.6) vs. −1.1% (SD 2.7), *p* = 0.55.

### 3.4. Inter Observer Reliability

In total, the LVEF and GLS of 20 patients were determined at baseline and 30-day follow-up by a second, blinded, observer. The ICC between the observers was 0.92 (95%CI: 0.84–0.96) for the LVEF measurements and 0.91 (95% CI: 0.81–0.95) for the GLS measurements.

## 4. Discussion

The main findings of the present analysis are: (1) There was no difference in improvement in LV function between patients randomized to an immediate (<3 h) or an early (12–24 h) invasive strategy, if 30-day follow up echocardiogram was compared to baseline. (2) LV function improved significantly in both groups with respect to EF and GLS recovery, if baseline was compared to 30-day follow-up echocardiogram.

Several trials have compared LVEF in NSTE-ACS patients according to (the timing of) treatment strategy. However, these trials are of heterogenic methodology, often not powered to detect a difference in LV function and did not use GLS to assess LV function. Tekin et al. compared an early invasive strategy (<24 h) to a delayed invasive strategy group (24–72 h) with respect to echocardiographic LVEF with a 3-month echocardiographic follow-up. A total of 131 NSTEMI patients participated in the study. Infarct-related arteries were equally divided between the study groups and the majority of patients in both groups had three-vessel disease. After 3 months, the prevalence of recurring MI, cardiac related hospitalization and death was significantly lower in the early invasive strategy group. While the discharge values of LVEF were similar (56.5% and 55.6%) between the two treatment strategies, the EF after 3 months in the early invasive group improved (59.3%) significantly more compared to the delayed invasive group (54.1%) [13]. The improvement of LVEF over time was in accordance with the findings of our study, whereas in our study, follow-up echocardiography was performed at 30 days instead of 3 months. The recent TRANSIENT trial evaluated an immediate (<0.5 h) intervention strategy with an early intervention strategy (<24 h) in 141 patients with a transient STEMI. Obviously, the pathophysiology of transient STEMI differs from NSTE-ACS. However, the two groups are randomized in a similar time frame in comparison to our study. In addition, infarct size is considered comparable to NSTEMI patients (mean of 10% of total myocardial mass, as measured by cardiac magnetic resonance (CMR)). The trial showed that an early intervention strategy was not inferior in terms of major adverse cardiovascular events (MACE) after 1 year. LVEF measured with CMR recovered similarly in both strategy groups after 4 months and did not differ between the study groups (immediate vs. delayed intervention). These findings are comparable to our analysis [14].

To the best of our knowledge, there are no studies comparing GLS outcomes for different timing strategies in NSTE-ACS patients. In line with our GLS results there is a study published by Grenne et al. that showed the deterioration of GLS in NSTE-ACS patients awaiting angiography. A total of 56 patients with NSTEMI were allocated to an early (<32 h) invasive strategy. Patients planned for very early (<10 h) strategy were excluded. Ultrasound GLS measurements were performed after admission and before angiography. The GLS values deteriorated significantly (from −16.1% ± 2.6% to −15.0% ± 2.6%) awaiting angiography, implying the progressive impairment of myocardial function by delaying an invasive strategy. The authors suggested benefit from immediate revascularization to relieve ischemia and prevent myocardial damage. However, after 3 months, follow-up GLS improved significantly compared to normal values (mean GLS −17.1% ± 3.1%) [15]. Since existing data of myocardial function and GLS in timing strategy studies using modern antiplatelet and anticoagulant therapy turns out to be scarce, we believe it is important to make the current study available for future researchers investigating the optimal timing of an invasive strategy in NSTE-ACS.

A plausible explanation for the improvement of EF and GLS function in both study groups in our current study is the effect of coronary revascularization itself and medical therapy allowing the recovery of myocardial stunning [16]. Striking is the association between NT-proBNP levels and LV function recovery. NT-proBNP is a well-known and widely accepted biomarker in risk assessment for NSTE-ACS patients. A possible explanation could be that that higher NT-proBNP levels at admission is an indicator of a greater amount of stunned myocardium that eventually recovered at the time of follow-up echocardiogram [1]. However, it is possible that at the time of the 30-day follow-up echocardiogram, a part of the myocardial cells are still in a state of hibernation [17]. To observe the full potential of myocardial recovery, one could consider choosing a longer period of time for the follow-up echocardiogram. However, the exact timing of an invasive strategy within the recommended 24-h time frame does not seem to influence the degree of LVF recovery.

### Limitations

Several limitations should be considered with respect to the current study. First, the current data is collected in a randomized controlled setting. However, the original study was powered to show a difference in AUC of CKMB. For this reason and the fact that the study was terminated early, it was not specifically powered to show a difference in EF and GLS. Second, several factors in the study design and baseline characteristics are limitations to the current sub-analysis: The study was single-center and had a long period of patient recruitment for a relatively small and heterogeneous study population. In addition, an outside core laboratory for analysis was not used. The reduction of EF at the initial hospitalization was mild for most patients. Patients were mostly male with low SYNTAX scores, and about 66% of patients had positive troponins. Third, 20% of echocardiographic data was not eligible for EF and/or GLS analysis. This was in most cases due to poor image quality, often in the semi-acute setting. It is not uncommon that part of the echocardiographic data is not suitable for analysis; lack of sufficient image quality to perform GLS in a non-acute clinical setting is reported in 7% of cases [18]. As our percentages are rather higher, this might have created a selection bias, since patients with poorer echocardiography windows, e.g., patients with higher BMI, might have been excluded. Fourth, several patients had a delay between diagnostic angiogram and revascularization treatment, such as CABG. We did not correct for this delay regarding the baseline echocardiogram nor when the follow-up echocardiogram was planned 30 days later. This could have influenced the amount of myocardial function recovery, although for the 30-day follow-up echocardiogram, one would expect most myocardial tissue to be recovered in the first weeks after the initial event [17].

## 5. Conclusions

In NSTE-ACS patients, if a direct (<3 h) invasive strategy was compared to an early (12–24 h) strategy, it did not result in a difference in LVEF or GLS recovery after 30 days. However, at 30-day follow-up, the LVF in both study groups showed a significant improvement in comparison to baseline echocardiography.

## Figures and Tables

**Table 1:** Baseline and procedural characteristics.

	Immediate (*n* = 110)	Early (*n* = 112)	*p* Value
Age, years	65.9 ± 10.5	65.8 ± 11.3	0.94 *
Gender, male	82 (75)	87 (78)	0.64
Body mass index, kg/m^2^	28.7 ± 4.5	27.6 ± 6.5	0.17 *
Duration of chest pain before admission, hours (IQR) †	3.0 (1.3–9.0)	4.0 (2.0–12.0)	0.18
ST depression > 0.1 mV or dynamic ST-segment changes	30 (27)	23 (21)	0.27
hsTroponinT > 1 ULN	81 (75)	82 (73)	0.73
GRACE-risk score ‡	116.0 ± 27.9	115.8 ± 28.3	0.96 *
Cardiac History			
Previous MI	27 (25)	24 (21)	0.63
Previous CABG	11 (10)	8 (7)	0.48
Previous PCI	25 (23)	26 (23)	1.00
Known congestive heart failure	1 (1)	1 (1)	1.00
Risk Factors			
Hypertension	58 (53)	48 (43)	0.14
Current smoking	40 (36)	43 (38)	0.89
Diabetes	28 (25)	18 (16)	0.10
Hypercholesterolemia	34 (31)	35 (31)	1.00
Positive family history	29 (26)	33 (29)	0.66
Peripheral artery disease	3 (3)	6 (5)	0.50
Age over 60 years	65 (59)	57 (51)	0.22
Medication administered in-hospital			
Aspirin ||	119 (95)	122 (98)	
Clopidogrel ||	9 (7)	5 (4)	
Prasugrel ||	3 (2)	2 (2)	
Ticagrelor ||	114 (91)	117 (94)	
Fondaparinux ||	112 (90)	117 (94)	
Glycoprotein IIb/IIIa inhibitors	0 (0)	0 (0)	
Angiography performed	110 (100)	112 (100)	1.00
Time to angiography, hours	3.0 (2.6–3.5)	22.9 (21.0–24.2)	<0.001 †
Syntax score	10.0 (4.5–21.0)	8.0 (4.0–16.3)	0.19 †
Number of diseased vessels ¶			0.07
0	13 (12)	12 (11)	
1	31 (28)	51 (46)	
2	32 (29)	25 (22)	
3	32 (29)	23 (21)	
Treatment §			0.11
PCI	59 (54)	75 (67)	
Ad-hoc procedure	56 (51)	67 (60)	
Staged procedure	3 (3)	8 (7)	
CABG	24 (22)	15 (13)	0.27 †
Delay from angiography, days	7.0 (3.0–10.0)	8.5 (5.8–13.3)	
Conservative	26 (24)	22 (20)	

Values are mean ± standard deviation (SD) or *n* (%), and the *p* values were calculated from the chi-square test unless listed otherwise. * Calculated from Student’s *t*-test. † Values are median (Interquartile range (IQR)), and *p* value was calculated from the Mann–Whitney test. ‡ GRACE-risk score: in-hospital death. || Given as pre-treatment before angiography. ¶ More than 50% narrowing by visual assessment in 3 orthogonal views. § Oral anticoagulation (vitamin K antagonist and/or NOAC) was an exclusion criterion. ULN = upper limit of normal, GRACE = global registry of acute coronary events; MI = myocardial infarction; CABG = coronary artery bypass graft; PCI = percutaneous coronary intervention.

**Table 2:** Comparison of Left Ventricular Ejection Fraction for ACS-patients.

	Immediate	Early	*p* Value
	(*n* = 85)	(*n* = 88)	
EF baseline, % (SD)	51.8 (9.6)	50.7 (8.8)	0.46
	(*n* = 90)	(*n* = 96)	
EF at 30-day FU, % (SD)	53.6 (8.6)	54.2 (7.9)	0.62
Improvement in EF:	(*n* = 73)	(*n* = 79)	
Follow-up-Baseline, % (SD)	2.5 (7.9)	3.3 (8.5)	0.51

Values are median (SD). The *p* values were calculated using the Student’s *t*-test and paired *t*-test. EF = ejection fraction; SD = standard deviation.

**Table 3:** Comparison of Left Ventricular Global Longitudinal Strain for ACS-patients.

	Immediate	Early	*p* Value
	(*n* = 88)	(*n* = 86)	
GLS baseline, % (SD)	−15.6 (3.3)	−16.3 (2.7)	0.17
	(*n* = 88)	(*n* = 95)	
GLS at 30-day FU, % (SD)	−16.4 (3.1)	−16.8 (2.8)	0.32
Improvement in GLS:	(*n* = 74)	(*n* = 77)	
Follow-up-Baseline, % (SD)	−0.8 (2.5)	−0.7 (2.8)	0.82

Values are median (SD). The *p* values were calculated using the Student’s *t*-test and paired *t*-test. GLS = global longitudinal strain, FU = follow-up; SD = standard deviation.

## Data Availability

The data presented in this study are available on request from the corresponding author. The data are not publicly available due to privacy reasons.

## Supplementary Materials

The following are available online at https://www.mdpi.com/article/10.3390/jcm10163636/s1, Supplementary file 1: Correlation between different variables and LVEF. Supplementary file 2: Final diagnosis in patients treated conservatively. Supplementary file 3: Comparison of change in ejection fraction and left ventricular global longitudinal strain for PCI-treated patients.
