# Supplementary material for: The Effect of the Timing of Invasive Management on Cardiac Function in Patients with NSTE-ACS, Insights from the OPTIMA-2 Randomized Controlled Trial
Source: J Clin Med. 2021 Aug 17;10(16):3636. doi: 10.3390/jcm10163636 (PMC8396935; doi:10.3390/jcm10163636)
Supplement: Supplementary file 1 [file jcm-10-03636-s001.zip › Supplementary File 1. Correlation Between different Variables and LVEF_v28-06.pdf]

**Supplementary file 1. Effect of different variables on overall Left Ventricular Function**		
	**ß-value Difference in EF after 30-days**	**p Value**
	**Total Population (n=152)**	
**Above 60 years of age**	-1.210 (-3.846-1.426)	0.37
**Diabetes Mellitus**	-1.088 (-4.209-2.033)	0.49
**Hypertension**	-2.318 (-4.942-0.306)	0.08
**Current Smoker**	-0.010 (-2.718-2.699)	0.99
**Hypercholesterolemia**	0.923 (-1.938-3.783)	0.53
**Peripheral arterial disease**	3.311 (-4.892-11.514)	0.43
**Positive Family History**	-0.239 (-3.121-2.643)	0.87
**GRACE score**	0.032 (-0.016-0.080)	0.18
**AUC CK-MB, ng/L/h**	0.000 (-0.001-0.001)	0.86
**AUC hs-TropT, ng/L/h**	0.011 (-0.027-0.049)	0.57
**NT-proBNP*, pmol/L**	0.021 (0.009-0.033)	<0.01
**hs-TropT*, ng/L**	-0.368 (-6.001-5.266)	0.90
**hsCRP*, mg/L**	0.033 (-0.089-0.155)	0.59
**NT-proBNP†, pmol/L**	0.016 (0.005-0.027)	<0.01
**Duration of Chest Pain, hours**	0.000 (0.000-0.000)	0.25
**Syntax score**	-0.063 (-0.202-0.075)	0.37
**Number of Diseased Vessels[Δ]**	-0.217 (-1.571-1.137)	0.75
**Treatment Strategy[¶]**	-0.096 (-2.631-0.669)	0.24
	**PCI Treated Patients only (n=97)**	
**Target vessel[ζ]**	1.437 (-0.583-3.458)	0.16
**Number of stents**	0.064 (-1.826-1.954)	0.95
	**ß-value Difference in GLS after 30-days**	**p Value**
	**Total Population (n=151)**	

**Above 60 years of age**	-0.505 (-1.356-0.347)	0.24
**Diabetes Mellitus**	-0.662 (-1.664-0.339)	0.19
**Hypertension**	-0.580 (-1.143-0.272)	0.18
**Current Smoker**	-0.473 (-1.353-0.407)	0.29
**Hypercholesterolemia**	-0.088 (-1.017-0.841)	0.85
**Peripheral arterial disease**	-1.089 (-3.730-1.553)	0.42
**Positive Family History**	0.078 (-0.846-1.0010)	0.87
**GRACE score**	-0.008 (-0.022-0.007)	0.29
**AUC CK-MB, ng/L/h**	0.000 (0.000-0.000)	0.99
**AUC hs-TropT, ng/L/h**	<0.01 (-0.003-0.022)	0.13
**NT-proBNP*, pmol/L**	0.007 (0.001-0.014)	0.03
**hs-TropT*, ng/L**	0.475 (-1.162-2.111)	0.57
**hsCRP*, mg/L**	0.011 (-0.026-0.049)	0.55
**NT-proBNP†, pmol/L**	-0.001 (-0.005-0.002)	0.45
**Duration of Chest Pain, hours**	0.000 (0.000-0.000)	0.18
**Syntax score**	-0.025 (-0.067-0.017)	0.25
**Number of Diseased Vessels**[Δ]	-0.348 (-0.779-0.082)	0.11
**Treatment Strategy**[¶]	-0.057 (-0.716-0.314)	0.34
	**PCI Treated Patients only (n=94)**	
**Target vessel**[ζ]	-0.232 (-0.864-0.400)	0.47
**Number of stents**	0.015 (-0.576 – 0.606)	0.96

Values are mentioned unit (95%CI). P value was calculated using univariate linear regression analysis. EF = Ejection Fraction; GLS = global longitudinal strain, FU = follow-up; SD = standard deviation; CI = confidence interval.

*** Admission Value † Discharge Value [Δ] >50% stenosis [¶] PCI, CABG or conservative ζ Distribution of target vessels: RCA 34 (25%), LAD 55 (41%), RCX 37 (28%), LM 1 (1%), SVG 3 (2%)**
