# Supplementary material for: The Effect of the Timing of Invasive Management on Cardiac Function in Patients with NSTE-ACS, Insights from the OPTIMA-2 Randomized Controlled Trial
Source: J Clin Med. 2021 Aug 17;10(16):3636. doi: 10.3390/jcm10163636 (PMC8396935; doi:10.3390/jcm10163636)
Supplement: Supplementary file 1 [file jcm-10-03636-s001.zip › Supplementary File 2. Diagnosis Conservative Treated_28-06.pdf]

Supplementary File 2. Final diagnosis of patients treated conservatively after initial angiography.

S2. Final diagnosis of patients treated conservatively after initial angiography

	**Immediate (n=41)**	**Early (n=33)**	**p Value**
**Diagnosis**			0.74
**MINOCA**	13 (32)	13 (39)	
**Culprit lesion(s) not suitable for PCI**	13 (32)	8 (24)	
**Tako-Tsubo Cardiomyopathy**	1 (2)	2 (6)	
**Myocarditis**	1 (2)	1 (3)	
**SCAD**	1 (2)	0 (0)	
**Type 2 NSTE-ACS**	0 (0)	1 (3)	
**Non-cardiac**	12 (29)	8 (24)	

The event rates, expressed as n (%), p-value was calculated with the use of the chi-square test.

MINOCA = myocardial infarction nonobstructive coronary arteries; PCI = Percutaneous Coronary Intervention; SCAD = spontaneous coronary artery dissection; NSTE-ACS = non-ST-segment elevation acute coronary syndrome
