# Supplementary material for: The Effect of the Timing of Invasive Management on Cardiac Function in Patients with NSTE-ACS, Insights from the OPTIMA-2 Randomized Controlled Trial
Source: J Clin Med. 2021 Aug 17;10(16):3636. doi: 10.3390/jcm10163636 (PMC8396935; doi:10.3390/jcm10163636)
Supplement: Supplementary file 1 [file jcm-10-03636-s001.zip › Supplementary File 3. PCI treated GLS and EF v28-06.pdf]

Supplementary file 3. Comparison of change in Ejection Fraction and Left Ventricular Global Longitudinal Strain for PCI treated patients

	Immediate (n=43)	Early (n=54)	p Value
**Improvement in EF: Follow-up-Baseline, % (SD)**	2.1 (8.1)	4.6 (8.8)	0.15
**Improvement in GLS: Follow-up-Baseline, % (SD)**	-0.8 (2.6)	-1.1 (2.7)	0.55

Values are median (SD). The p Values were calculated using the student t-test and paired t-test. GLS = global longitudinal strain, FU = follow-up; SD = standard deviation
